# Breast Ultrasound Images Augmentation and Segmentation Using GAN with Identity Block and Modified U-Net 3+

**DOI:** 10.3390/s23208599

**Published:** 2023-10-20

**Authors:** Meshrif Alruily, Wael Said, Ayman Mohamed Mostafa, Mohamed Ezz, Mahmoud Elmezain

**Affiliations:** 1 College of Computer and Information Sciences, Jouf University, Sakaka 72388, Saudi Arabia; mfalruily@ju.edu.sa (M.A.); maismail@ju.edu.sa (M.E.); 2 Computer Science Department, Faculty of Computers and Informatics, Zagazig University, Zagazig 44511, Egypt; wael.mohamed@zu.edu.eg; 3 Computer Science Department, College of Computer Science and Engineering, Taibah University, Medina 42353, Saudi Arabia; 4 Computer Science Department, Faculty of Science, Tanta University, Tanta 31527, Egypt; mmahmoudelmezain@taibahu.edu.sa; 5 Computer Science Department, College of Computer Science and Engineering, Taibah University, Yanbu 966144, Saudi Arabia

**Keywords:** ultrasounds, breast cancer, augmentation, segmentation, U-Net 3+

## Abstract

One of the most prevalent diseases affecting women in recent years is breast cancer. Early breast cancer detection can help in the treatment, lower the infection risk, and worsen the results. This paper presents a hybrid approach for augmentation and segmenting breast cancer. The framework contains two main stages: augmentation and segmentation of ultrasound images. The augmentation of the ultrasounds is applied using generative adversarial networks (GAN) with nonlinear identity block, label smoothing, and a new loss function. The segmentation of the ultrasounds applied a modified U-Net 3+. The hybrid approach achieves efficient results in the segmentation and augmentation steps compared with the other available methods for the same task. The modified version of the GAN with the nonlinear identity block overcomes different types of modified GAN in the ultrasound augmentation process, such as speckle GAN, UltraGAN, and deep convolutional GAN. The modified U-Net 3+ also overcomes the different architectures of U-Nets in the segmentation process. The GAN with nonlinear identity blocks achieved an inception score of 14.32 and a Fréchet inception distance of 41.86 in the augmenting process. The GAN with identity achieves a smaller value in Fréchet inception distance (FID) and a bigger value in inception score; these results prove the model’s efficiency compared with other versions of GAN in the augmentation process. The modified U-Net 3+ architecture achieved a Dice Score of 95.49% and an Accuracy of 95.67%.

## 1. Introduction

One of the diseases that both men and women can be affected by is breast cancer, which can affect women in particular. Recently, with the spread of this dreaded disease and the infection of a large number of women around the world, the concerned groups had to find ways to detect and segment this disease at an early stage to help doctors diagnose it and start the treatment journey early, whether with chemotherapy or surgical intervention [1,2,3,4,5]. With the onset of the disease, many methods and models were introduced to detect and segment the tumor, such as machine learning and deep learning methods [6,7]. In particular, deep learning provided many models for the discovery and dissection of disease, such as U-Net, convolution neural network (CNN), and fully convolution neural network (FCN). Each of them proved their competence in detecting and segmenting the disease and diagnosing it quickly in accurate ways and with high accuracy.

Preprocessing before the segmentation process can support increasing the accuracy of the segmentation process. There are different types of preprocessing of segmentation, such as removing noise from the ultrasounds and augmenting the dataset for use in the training process. The augmentation process can also assist in increasing the dataset sample size. Incorporating more samples into the dataset improves the training efficiency and segmentation precision [8,9].

Recently, due to the improved performance in ultrasonic augmentation compared to several old and classical approaches, CNN has grown increasingly as one of the major deep learning models [10,11]. To succeed, CNNs need big datasets or large numbers of training images to produce highly accurate segmentation results, which are particularly scarce in the medical field due to the high cost of medical imaging. In order to produce a large number of training sets from the available ultrasounds without needing to do new ultrasounds, the augmentation process is used. GAN introduce a highly efficient method for augmenting new ultrasound images from already produced ultrasounds. Many versions of GAN have been introduced in the augmentation process, but the main challenge is dealing with the mode collapse problem and the smoothing function. Mode collapse occurs when the generator part generates only a few different samples that deceive the discriminator instead of creating a varied collection of samples that span the whole target distribution. This can occur when the discriminator becomes too strong and can recognize and exploit patterns in the generator’s output, causing the generator to focus solely on creating those patterns instead of investigating the whole range of possible outputs. This paper presents some modifications to reduce mode collapse during augmentation. Enlarging the ultrasound dataset efficiently can help the segmentation model increase the accuracy of the segmentation process [12,13,14].

Deep learning has introduced many architectures for segmenting ultrasounds efficiently after the argumentation process, such as the fully convolutional network (FCN), CNN, the artificial neural network (ANN), and U-Net [15]. The CNNs rely on classifying ultrasounds, where the image is treated as input, and the output is generated as a single label. However, they are ineffective in biomedical scenarios, particularly when segmenting medical images like ultrasounds. U-Net was introduced to address this issue. Many architectures of U-Net have been introduced in ultrasound segmentation, such as attention U-Net, residual U-Net, and attention residual U-Net [16,17,18]. The U-Net 3+ architecture is a modified version of the U-Net that was introduced using full-scale skip connections [19]. Full-scale feature fusion, on the other hand, might result in unduly redundant calculations. The main goal of using U-Net 3+ was to decrease the network while boosting the feature extraction capabilities. To begin, the model prunes the full-scale skip connections of U-Net 3+ to eliminate duplication and increase computational efficiency. U-Net 3+ redesigns the skip connections to incorporate full-scale semantic information from the input photos. It showed that it was not correct but quicker and more effective than several common image segmentation architectures. This paper proposes the following items:

1. The modified GAN with identity solve the mode collapse problem in the other versions of GAN for argumentation with ultrasounds.
2. The framework uses the identity block with GAN and a modified loss function for expanding the ultrasound dataset.
3. The argumentation process for the ultrasounds helps increase the segmentation process’s accuracy for different U-Net architectures.
4. The combination of the GAN with identity blocks and the U-Net 3+ achieves higher accuracy when compared with other models in both the argumentation and segmentation processes.

The paper provides an introduction and an overview in Section 1 and Section 2 about the segmentation and argumentation of ultrasound images for detecting breast tumors. Section 3 introduces the identity GAN in the augmentation step and the U-Net 3+ in the segmentation process. The experimental findings are presented in Section 4, while Section 5 provides the conclusion and the recommended future work.

## 2. Related Work

The segmentation process using deep models greatly impacts the medical field, especially in breast tumor segmentation using U-Net. Many architectures of U-Net have been introduced for the segmentation process, such as U-Net [20], U-Net ++ [21], Attention U-Net [21,22], Vanilla U-Net [22,23], and Stack U-Net [24]. Although all the previously mentioned methods are useful in detecting cancerous breast tumors and other tumors, these methods find themselves restricted and insufficient when the dataset is small, and this causes poor accuracy of the results. For solving the problem of the small dataset, the GAN model presents many solutions for the general augmentation of medical images and breast ultrasounds [25]. Maria et al. [26] introduced a modified version of the GAN called the UltraGAN; the modified model by the authors was used to increase the number of ultrasound images using quality transfer while preserving structural information. UltraGAN employs frequency loss functions and an anatomical coherence restriction to improve quality. We demonstrate image quality enhancement without losing anatomical consistency. Using a publicly available dataset, we verify UltraGAN for echocardiogram segmentation and show that quality-enhanced pictures can benefit downstream tasks. We share our source code and training models to ensure repeatability. Miomoria et al. [27] used a deep convolution generative adversarial network (DCGAN) in ultrasound generation to enhance the segmentation process and increase the number of ultrasound images. In order to generate novel synthetic images comparable to the actual images, DCGAN uses CNNs to learn a hierarchy of characteristics from the input images. The discriminator network can differentiate between authentic and fraudulent images, while the generator network creates an image from a random noise vector as input.

DCGAN will employ CNNs to learn a hierarchy of characteristics from input photos, producing new, synthetic images comparable to genuine ones. The discriminator network distinguishes between authentic and fake images, whereas the generator network constructs an image from an additive noise vector. The authors use 528 ultrasound samples for the breast, divided into 144 benign samples and 529 ultrasounds of 216 malignant samples, and they generate using a DCGAN with 50, 100, 200, 500, and 1000 epochs. Robust data augmentation GAN (RDAGAAN) [28] are a modified GAN model also introduced for augmenting limited datasets before medical segmentation or object detection, such as tumor detection. The authors combined several small datasets from multiple sources. Creating an image is then split into two networks: one for object generation and the other for the translation of the image. The image translation network combines the images generated from the network with the images contained within the bounding boxes of the input dataset. A quantitative investigation revealed that the produced photos increased the detection ability of the YOLO v5 model. A modified version of GAN called stacked generative adversarial networks (StackGAN) [29] can be utilized to invent new samples of images from a smaller dataset or image deduction. Despite its ability to generate high-resolution images, particularly ultrasound and brain images, it has several drawbacks, including the tendency for some images to be incoherent and the possibility of mode collapse.

Lennart et al. [30] established a novel architecture for augmenting synthetic ultrasounds with speckle noise using generative adversarial networks (GAN). The key component is a speckle layer, which may be added to a neural network to produce real and domain-specific speckles. The generated GAN architecture is known as the speckle GAN. Speckle GAN’s discriminator network is taught to discriminate between actual and manufactured speckle patterns. Speckle GAN may produce synthetic speckle patterns that are very realistic and visually indistinguishable from actual speckle patterns by tuning the generator and discriminator networks in an adversarial way.

Although many models and methods have been used in both segmentation and augmentation, all of them have their limitations, such as the accuracy of the segmentation process, the mode of collapse in the generation of the ultrasound samples, and the loss function.

## 3. Proposed Model

This section of the paper introduces the methodology of the hybrid approach for segmentation and detecting the tumor from ultrasound images. The hybrid approach starts by importing an ultrasound dataset containing ultrasounds for women between 25 and 75 for 600 female patients in all. The dataset consists of 780 images, each measuring 500 by 500 pixels, and the images are categorized into normal, benign, and malignant. The dataset contains 487, 210, and 133 for benign, malignant, and normal, respectively. The second step is about augmenting the dataset using the modified GAN, and the last step is about segmentation and detecting the breast tumor using the U-Net 3+.

The U-Net 3+ architecture is an advanced modification of the U-Net model, specifically designed for image segmentation tasks. It comprises three main components: the encoder, bottleneck, and decoder, each playing a crucial role in the overall architecture. The encoder is responsible for capturing high-level semantic information from the input image. It consists of a series of convolutional layers that progressively reduce the spatial dimensions while increasing the number of feature channels. These convolutional layers typically incorporate operations such as convolution, batch normalization, and non-linear activation functions like ReLU. By applying these operations, the encoder extracts abstract features that represent the underlying structures and patterns in the input image.

The bottleneck component serves as the central part of the U-Net 3+ architecture. It typically consists of multiple convolutional layers with a smaller number of filters compared to the encoder layers. The purpose of the bottleneck is to compress the encoded features into a compact representation, also known as a latent space. This compression helps retain the most relevant information necessary for accurate segmentation while reducing the computational complexity of the model. The decoder is responsible for upsampling the compact representation from the bottleneck and recovering the spatial dimensions to match the original input size. Similar to the encoder, the decoder consists of convolutional layers, batch normalization, and activation functions. However, what sets the U-Net 3+ decoder apart is the incorporation of skip connections. The skip connections establish connections between corresponding layers in the encoder and decoder paths. These connections allow the network to preserve and utilize fine-grained details from the encoder during the upsampling process. Specifically, the skip connections concatenate feature maps from the encoder to the corresponding decoder layers, enabling the decoder to access both low-level and high-level features. This integration of fine-grained details helps in the precise localization and segmentation of objects in the image.

**Figure 1:**
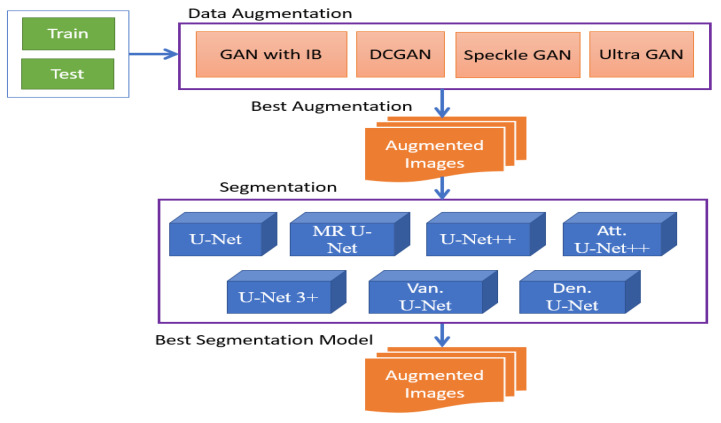
Methodology block diagram.

In the U-Net 3+ architecture, multiple decoder paths with different scales or resolutions of skip connections are employed. This multi-scale design allows the network to capture features at various levels of abstraction and combine information from different scales effectively. By considering features at multiple scales, the U-Net 3+ architecture enhances the segmentation process, enabling more accurate and detailed segmentation results. Figure 1 shows the block diagram of the hybrid approach.

### 3.1. GAN with Identity Block

Generative adversarial networks (GAN) exhibit a set of exceptional qualities that make them exceptionally well-suited for enhancing image processing tasks. Firstly, their generative capability allows them to generate new images that closely resemble real ones, enabling improvements in various aspects of image quality. GAN can capture intricate details, textures, and structures present in the training data, resulting in visually appealing and high-quality images. Secondly, GAN employ an adversarial training framework where a generator network and a discriminator network compete against each other. The generator learns to produce images that are indistinguishable from real images, while the discriminator aims to correctly classify real and generated images. This adversarial process drives the generator to continually improve its image-generation abilities, leading to the production of more realistic and visually pleasing results. Furthermore, GAN excel at learning complex non-linear transformations between input and output images.

Traditional linear processing methods often struggle to capture intricate relationships and patterns in complex image data. However, GAN can learn and represent these non-linear mappings, enabling them to enhance various image attributes effectively. This includes improving resolution, enhancing sharpness, adjusting color balance, and refining fine details that are challenging to achieve with traditional linear techniques. Another key advantage of GAN is their ability to learn from unannotated or partially annotated data. This unsupervised or self-supervised learning capability is particularly valuable in image processing tasks where obtaining large-scale annotated datasets can be time-consuming and expensive. GAN can leverage the inherent patterns and structures in the training data to enhance images without relying on explicit labels or annotations.

Lastly, GAN offer flexibility and adaptability to cater to specific image processing goals. They can be customized by modifying the network architecture, loss functions, or training strategies to suit the desired enhancements. This adaptability allows GAN to tackle a wide range of image processing tasks, including but not limited to super-resolution, denoising, deblurring, style transfer, and inpainting.

The architecture of the GAN with identity block (IGAN) contains two main parts, as in traditional GAN: the first part includes the generator, while the second includes the discriminator. The GAN with identity block model uses the proximity of every pair of samples in a single mini-batch. Then, the overall summary of a single data point is computed by adding its proximity to other samples in the same batch. Finally, it is explicitly added to the model. The discriminator is still required to output a single number for each example. This number indicates the likelihood of the example originating from the training data. The single output of the discriminator with mini-batch training is allowed to use other examples in the mini-batch as side information. The model presents a solution to the problem of mode collapses in the discriminator, such as the Softmax function or the Gumbel-Softmax function that introduces randomness into the generator’s output, making it more difficult for the discriminator to identify patterns and forcing the generator to explore a wider range of possible outputs. The GAN with identity blocks changes the loss function from Equation (1) to avoid mode collapse by adding smoothing in the other versions of the GAN during the image generation in the augmentation process.

**Figure 2:**
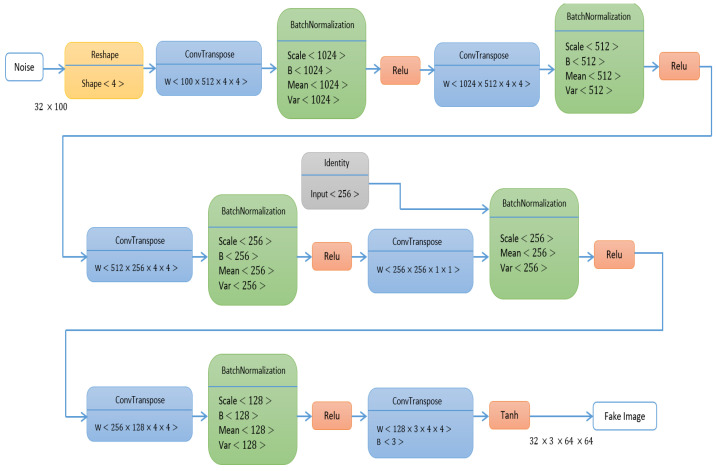
IGAN generator architecture.

**Figure 3:**
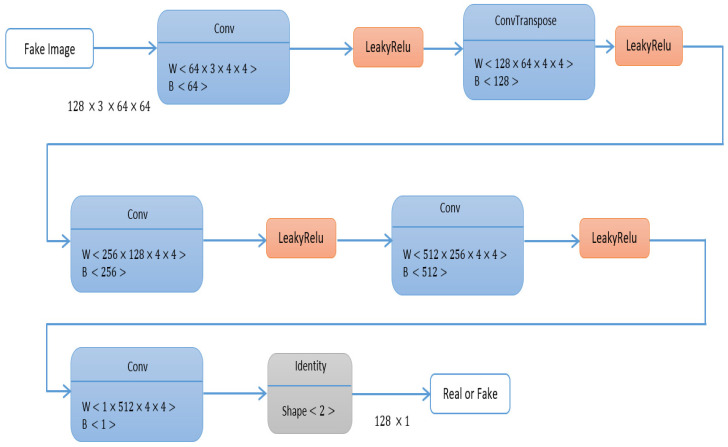
IGAN discriminator architecture.

Mode collapse occurs when the discriminator returns one or zero as the classification result for the real and fake images. Gradients will be close to zero at both ends, and the discriminator cannot provide useful feedback, resulting in a vanishing gradient problem. As a result, this study alters the GAN loss function stated in Equation (1) by including label smoothing. Instead of supplying 1 and 0 labels for real and false data while training the discriminator, we used 0.9 and 0.1. Equations (2)–(4) express the loss function employed in this study. Figure 2 explains the architecture of the generator, and Figure 3 explains the architecture of the discriminator.

(1) $$\underset{G}{\min}\underset{S}{\max}E_{x∼q_{\mathrm{data}\;}\left(x\right)}\left[\log S\left(x\right)\right]+E_{z∼p\left(z\right)}\left[\log\left(1-S\left(G\left(z\right)\right)\right)\right]$$

(2) $$L\left(G,S\right)=\min\left(L_{G}\right)+\max\left(L_{S}\right)$$

(3) $$L_{G}=-\frac{1}{m}\sum_{i=1}^{m}\left[\log S\left(G\left(z\right)\right)\right]\;\;$$

(4) $$L_{S}=\frac{1}{m}\sum_{i=1}^{m}\left[\log S\left(x\right)+\log\left(1-S\left(G\left(z\right)\right)\right)\right]$$

The GAN with the identity block begins by importing a limited ultrasound dataset, loading the ultrasound from the patients in the dataset, and then extracting the semantic label from the lesion area and from the contour, also synthesizing the semantic label. The main vital stage in the augmentation is feeding the tumor with the generator to obtain the synthesized image and calculating the regional loss, as mentioned in the previous subsection. The architecture was trained using an optimizer called Adamx that uses the gradient clipping technique to limit gradients’ size to prevent noisy updates. The learning rate was set to 0.0005 with a batch size of 256.

### 3.2. Evaluation Metrics

The framework uses two different evaluation metrics for comparing the results of the identity GAN and another version of GAN. The framework uses Fréchet inception distance (FID) and inception score (IS) using Equations (5) and (6). In Equation (5), *x* represents a single generated sample drawn from the generator, while y corresponds to the label prediction obtained through the Inception model. The FID and IS are introduced as metrics for evaluating the quality and similarity of images generated by generative adversarial networks (GAN).

The inception distance is computed using the Inception v3 neural network, which was trained for classifying images using the ImageNet dataset. The basic idea is to use the Inception network for feature extraction from both the real and generated images and then compare the distribution of these features using the Kullback–Leibler (KL) divergence. The FID modifies the inception distance by comparing the feature distributions of the real and generated images using the Fréchet distance, which measures the distance between two multivariate Gaussian distributions. The *r* in Equation (6) refers to the real image, while g denotes the generated image. The paper also uses Precision, Recall, Accuracy, and Dice Score for model evaluation using Equations (7)–(10), respectively.

(5) $$IS=exp\left(E_{x\sim p_{g}}D_{KL}\left(p\left(y|x\right)||p\left(y\right)\right)\right)$$

(6) $$FID=||\mu_{r}-\mu_{g}\;{\left|\right|}^{2}+Tr\;(\Sigma r+\Sigma g-2\;\sqrt{\left(\Sigma r\Sigma g\right)})\;\;$$

(7) $$Precision=\frac{{TP}_{i}}{{TP}_{i}+{FP}_{i}}\times 100\%$$

(8) $$Recall=\;\frac{{TP}_{i}}{{TP}_{i}+{FN}_{i}}\times 100\%$$

(9) $$Accuracy=\frac{{TP}_{i}+{TN}_{i}}{{TP}_{i}+{TN}_{i}+{FP}_{i}+{FN}_{i}}\times 100\%$$

(10) $$Dice\;Score=\frac{2\times |Precision\times Recall|}{\left|Precision+Recall\right|}\times 100\%$$

The FID is computed on a large number of samples from both the real and generated datasets, typically 10,000 and more, in order to obtain an accurate estimate of the distance between their distributions. In general, lower values of ID and FID indicate better quality of the generated images, as they imply a closer match between the distribution of features in the real and generated images.

### 3.3. The Modified U-Net 3+

In order to detect the breast tumor, the framework segments the ultrasounds using the U-Net 3+ architecture. The U-Net applied the same architecture but with a redesigned skip connection. The new skip connections consider the beneficial effect of particular multi-scale feature maps on segmentation. Each decoder layer in U-Net 3+ will have neighboring multi-scale feature maps that contribute similarly to segmentation, resulting in unnecessary duplicate calculations. The model has three modifications when compared with the previous versions of U-Net and U-Net ++.

The first modification is based on adopting and updating its deep supervision approach to accept full-scale semantic information. The second modification is based on modifying the dense connection architecture to cater to both low and high degrees of information in feature maps more efficiently for segmentation. The last modification is used to lower false-positive rates by employing a categorization-guided module to anticipate items when they are not there. The model uses a loss function that contains the summation of the focal loss ($f_{1}$), multi-scale structural similarity index loss (ms−ssim), and intersection over union (IoU) loss. Equation (11) shows the loss function of the modified U-Net 3+.

(11) $$l_{seg}=l_{f1}+l_{ms-ssim}+l_{IoU}$$

The model uses the convolution block attention module (CBAM) to enhance the architecture’s capacity for feature extraction. CBAM is a light feed-forward neural convolutional neural network attention module that can be implemented into any CNN architecture for end-to-end training and is one of several attention models. The CBAM modules contain two main blocks: the channel attention module is the first, and the spatial attention module is the second. The two channels are used to enhance the process of feature extraction. The model was trained with 200 epochs, a 0.0001 learning rate, and the modified loss function with Adamx as the optimizer.

### 3.4. The Dataset

**Figure 4:**
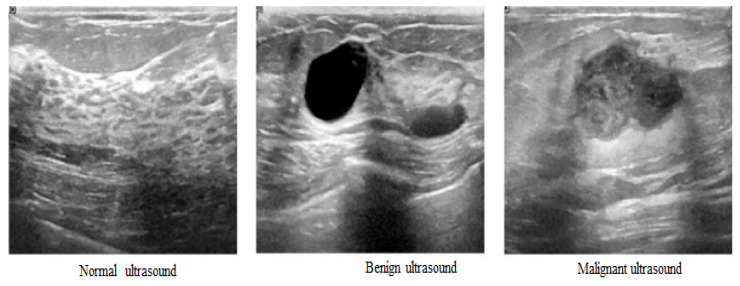
Normal, benign, and malignant ultrasound.

Women between the ages of 25 and 75 provided breast ultrasound scans for the study’s initial collection. These data were compiled in 2018 with 600 total female patients. Each of the 780 images in the dataset is 500 by 500 pixels. PNG files are used for the images. The original photos are shown with the ground truth photographs. The photos are divided into three categories: normal, benign, and malignant, as shown in Figure 4. The dataset still needs to be improved due to the small number of patients. The breast ultrasound images dataset [31], which is publicly available, has been obtained from Kaggle’s data repository [32].

### 3.5. Hardware and Software Specifications 

This paper is based on processor dual-core Intel(R) i7-11700, NVIDIA GEFORCE RTX 3080 GPU with random access memory, and Python programming languages in all experiments such as augmentation and segmentation process.

## 4. Results and Discussion

The experiments on ultrasonic augmentation using the GAN with identity (IGAN) and the findings of ultrasound segmentation using the U-Net 3+ are presented in this section. The section also compares IGAN, DCGAN, UltraGAN, and speckle GAN based on FID and IS. The section also compares segmentation using different architectures of U-Net and the four augmentation models. 

**Figure 5:**
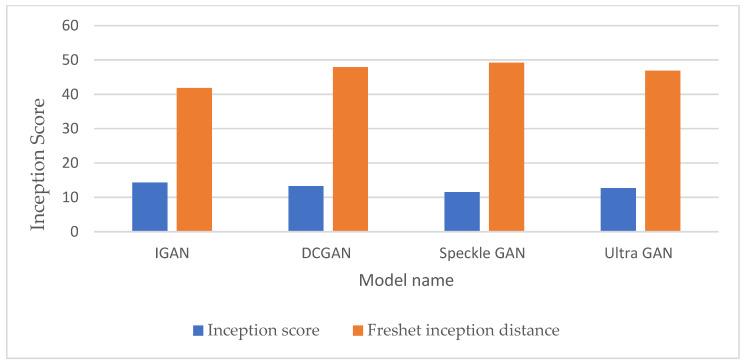
Augmentation results comparison.

Table 1 provides a comparison of IGAN with DCGAN [33], speckle GAN [30], and UltraGAN [26] regarding IS and FID. These comparisons were made after 200 epochs of training, using a learning rate of 0.0001 and the Adam optimizer. The results in Table 1 show the efficiency of IGAN compared with other available architectures in the augmentation process due to the larger IS and smaller FID. The evaluation in Table 1 was conducted using Fréchet’s inception distance and inception score using Equations (6) and (7). Figure 5 additionally presents a comparison of IGAN with other GAN architectures in terms of the augmentation process. The IGAN model yields a 7.75% improvement in IS compared to the best results achieved with DCGAN, while it provides a 10.77% enhancement in FID compared to the top-performing results obtained by UltraGAN.

Enhancing the accuracy of ultrasound breast segmentation can be achieved by leveraging different versions of GAN in the augmentation process. The choice of GAN architecture, loss functions, and training parameters can significantly impact the quality and diversity of the generated ultrasound images and also participate in increasing the accuracy of the segmentation process. The next tables discuss the results of segmentation after using the augmentation process using GAN and also show comparisons between the segmentation accuracy after using different versions of GAN.

Table 2 shows the results of the ultrasound segmentation before using any augmentation models. The table shows the efficiency of U-Net 3+ when compared with other U-Net architectures. All the U-Net architectures in the table use Adamx as an optimizer, 0.0001 as a learning rate, and 128 as a batch size. The experiments have been conducted using the same hardware specification. The last column in the table introduces the time per epoch in minutes. The U-Net 3+ model achieves scores of 92.68, 92.68, 92.67, 92.36, and 24.65 for Dice Score, Accuracy, Precision, and Recall, respectively, as well as 24.65 min per epoch without applying any augmentation preprocessing.

Hyperparameter optimization is a crucial step in developing effective U-Net architectures for tumor segmentation. The choice of optimizer and learning rate are key hyperparameters that significantly impact the model’s performance. In the case of the mentioned U-Net architectures, Adamx is specifically selected as the optimizer, providing adaptive learning rates and momentum to enhance convergence speed and generalization. The learning rate of 0.0001 strikes a balance between convergence speed and stability, allowing the model to effectively adjust its parameters during training. However, it is important to note that these hyperparameter values should be fine-tuned through systematic experimentation and evaluation to identify the optimal combination for the specific tumor segmentation task.

Techniques such as grid search, random search, Bayesian optimization, or automated hyperparameter tuning libraries can be employed to explore the hyperparameter space and uncover the best configuration. Cross-validation is commonly used to assess the model’s performance across different hyperparameter settings. By carefully optimizing these hyperparameters, researchers and practitioners can ensure that the U-Net architectures perform optimally and deliver accurate tumor segmentation results.

Table 3 showcases the results of ultrasound breast image segmentation using DCGAN exclusively as a pre-augmentation method. The information in Table 3 highlights the enhancements in segmentation performance, which encompass improvements in Dice Score, Accuracy, Precision, and Recall, achieved through the integration of the DCGAN architecture. Notably, these enhancements occur with a limited scaling factor. The impact of DCGAN is an increase of 0.84%, 0.98%, 0.85%, and 1.29% for Dice Score, Accuracy, Precision, and Recall, respectively.

Table 4 shows the segmentation after augmentation using a StackGAN. The table shows the enhancement of the segmentation results in terms of Dice Score, Accuracy, Precision, and Recall after using the augmentation using only the StackGAN architecture. The effect of StackGan is evident in an increase of 0.84%, 1.08%, 1.32%, and 1.28% in terms of Dice Score, Accuracy, Precision, and Recall, respectively. The enhancement occurs with a small-scale adjustment, a little increase to the DCGAN.

Table 5 shows the segmentation of ultrasound breast images after using the UltraGAN as a pre-augmentation process. The data presented in Table 5 also signify an increase of 0.84%, 1.07%, 1.29%, and 1.76% for the selected evaluation metrics. Similar to the previous DCGAN and StackGAN, the UltraGAN model also introduces a limited scaling factor.

Table 6 shows the outcomes of ultrasound image segmentation using the GAN with an identity block (IGAN) model. The data in Table 6 emphasize the efficiency of IGAN when compared to other GAN architectures, specifically DCGAN, StackGAN, and UltraGAN. This efficiency is represented by an increase of 3.03% for Dice Score, 3.23% for Accuracy, 3.15% for Precision, and 3.59% for Recall. The results illustrate the significant impact of using IGAN as a preprocessing step for the augmentation process.

**Figure 6:**
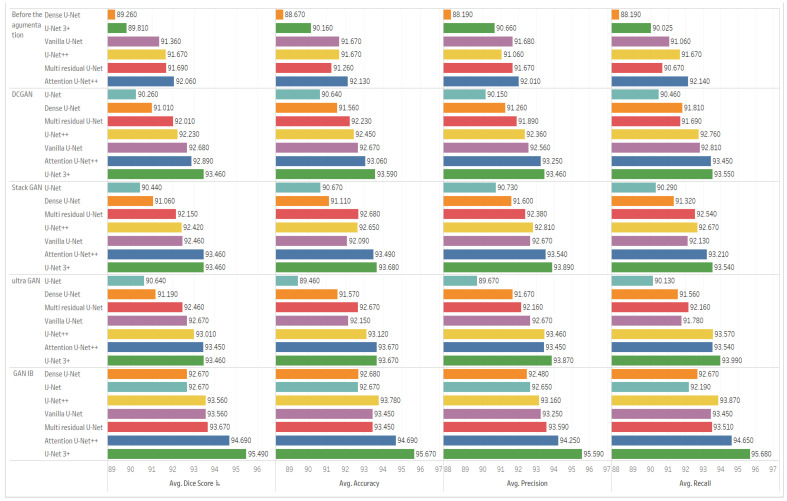
Comparison between different architectures of U-Net before and after augmentation.

Figure 6 shows the comparison between different architectures of U-Net before and after augmentation. Figure 6 is divided into five sections, each comparing the various U-Net architectures regarding Dice Score, Accuracy, Precision, and Recall. The first section of the figure shows the comparison before the augmentation process. The second section or row compares the architectures after using the DCGAN in the augmentation. The third section shows the comparison after using the speckle GAN, the fourth section shows the comparison after using the UltraGAN, and the last section shows the comparison after using the GAN with identity block. The results in the last section show the efficiency of the U-Net 3+ and GAN with identity blocking compared to all other sections in the figure.

**Figure 7:**
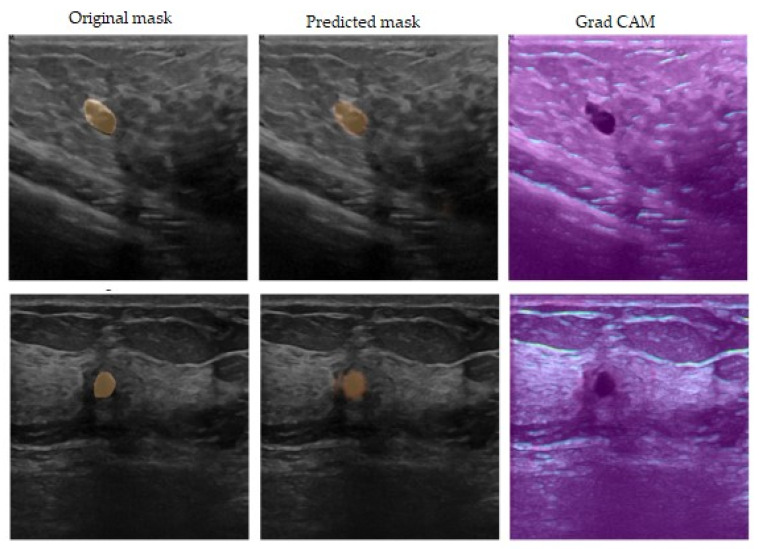
Original mask, predicted mask, and GRAD-CAM.

Figure 7 shows the segmentation results after using the GAN with an identity block and U-Net 3+. The figure is divided into three sections: the first column shows the input ultrasound image, the second column shows the predicted mask for the breast tumor, and the last column explores the use of the GRAD-CAM algorithm to provide a clearer understanding of the proposed model’s decision-making process.

The results in the previous tables show the efficient enhancement of using the combination of different architectures of U-Nets and different versions of GAN in augmentation, and it is important to note that the success of this combined approach depends on the quality and diversity of the augmented data generated by GAN. The U-Net 3+ architecture is well-suited for medical image segmentation tasks with its encoder–decoder structure and skip connections. It can effectively capture both local and global contextual information in the images, enabling accurate segmentation of structures of interest. The combination of identity GAN augmentation and U-Net 3+ leverages the strengths of both approaches, allowing for a more robust and accurate segmentation model when compared with other combinations between different architectures of U-Net and other versions of GAN for the augmentation process, as mentioned in Table 3, Table 4, Table 5 and Table 6. So, after different experiments and using different versions of modified GAN for augmentation, the results prove the efficiency of using different GAN in enhancing the accuracy of the segmentation process and also prove the efficiency of GAN with identity blocks in enhancing different architectures of U-Nets. The results also prove that the combination between U-Net 3+ and GAN with identity blocks is a well-situated combination among other combinations.

The proposed combination model, which incorporates both a GAN with an identity block and a U-Net 3+ architecture, is not limited to the detection of breast cancer from ultrasound images. Given that ultrasound images can be used to visualize blood flow and detect clots within blood vessels, we could apply the proposed combination model for blood coagulation detection. This combination allows us to leverage the generative capabilities of the GAN to generate synthetic coagulation images and then use the U-Net 3+ model for precise segmentation and detection.

## 5. Conclusions and Future Work

This paper provided a hybrid method for segmenting breast tumors from an ultrasound dataset. The model presented two main stages: the first is using the GAN with identity blocks for the augmentation process, and the second is segmenting the ultrasound images to detect the tumor. The combination of GAN with identity block for augmentation and U-Net 3+ architecture has proven effective enhancement in the segmentation accuracy of breast ultrasound images. The modified GAN with nonlinear identity blocks overcame different types of modified GAN in the ultrasound augmentation process, such as speckle GAN, UltraGAN, and deep convolutional GAN. The modified U-Net 3+ also overcame different architectures of U-Nets in the segmentation process. The proposed combination of results achieved a Dice Score of 95.49, an Accuracy of 95.67, a Precision of 95.59, and a Recall of 95.68.

The GAN model with identity block provides a powerful framework for data augmentation, generating synthetic images that closely resemble real data while introducing variations and diversity, solving the mode collapse, and vanishing gradient problems. The experiments also prove the efficient enhancement of different types of U-Net architectures and the efficiency of using GAN with identity blocks compared with other versions of GAN in the augmentation step. The results also prove the efficiency of U-Net 3+ with modified GAN when compared with other architectures of U-Net with identity blocks. Finally, GAN with identity blocks can effectively increase the medical datasets, which also helps increase the accuracy of different types of medical segmentation, regardless of the type of medical image or the different characteristics of U-Nets. Despite achieving these positive results, some challenges still exist, such as generating high-quality medical images. In order to bridge the gap between research and real-world applications, we intend to collaborate with clinical experts to conduct clinical validation studies, ensuring that our architecture meets the standards and requirements of the medical field.

## Figures and Tables

**Table 1:** Comparative analysis of evaluation metrics for IGAN-generated images.

Model	IGAN	DCGAN	Speckle GAN	UltraGAN
Inception Score	14.32	13.29	11.53	12.68
Fréchet Inception Distance	41.86	47.9	49.2	46.91

**Table 2:** A pre-augmentation comparative of different segmentation models.

Model	Dice Score	Accuracy	Precision	Recall	Time per Epoch (min)
U-Net [34]	86.94	87.64	88.65	87.69	24.26
Multi residual U-Net [35]	91.69	91.26	91.67	90.67	24.65
U-Net++ [36]	91.67	91.67	91.06	91.67	25.23
Attention U-Net++ [37]	92.06	92.13	92.01	92.14	24.36
Vanilla U-Net [38]	91.36	91.67	91.68	91.06	27.23
Dense U-Net [39]	89.26	88.67	88.19	88.19	24.67
U-Net 3+	92.68	92.68	92.67	92.36	24.65

**Table 3:** A comparative of different segmentation models using only DCGAN pre-augmentation.

Model	Dice Score	Accuracy	Precision	Recall
U-Net	90.26	90.64	90.15	90.46
Multi residual U-Net	92.01	92.23	91.89	91.69
U-Net++	92.23	92.45	92.36	92.76
Attention U-Net++	92.89	93.06	93.25	93.45
Vanilla U-Net	92.68	92.67	92.56	92.81
Dense U-Net	91.01	91.56	91.26	91.81
U-Net 3+	93.46	93.59	93.46	93.55

**Table 4:** A comparative of different segmentation models using only StackGAN pre-augmentation.

Model	Dice Score	Accuracy	Precision	Recall
U-Net	90.44	90.67	90.73	90.29
Multi residual U-Net	92.15	92.68	92.38	92.54
U-Net++	92.42	92.65	92.81	92.67
Attention U-Net++	93.46	93.49	93.54	93.21
Vanilla U-Net	92.46	92.09	92.67	92.13
Dense U-Net	91.06	91.11	91.60	91.32
U-Net 3+	93.46	93.68	93.89	93.54

**Table 5:** A comparative of different segmentation models using only UltrGAN pre-augmentation.

Model	Dice Score	Accuracy	Precision	Recall
U-Net	90.64	89.46	89.67	90.13
Multi residual U-Net	92.46	92.67	92.16	92.16
U-Net++	93.01	93.12	93.46	93.57
Attention U-Net++	93.45	93.67	93.45	93.54
Vanilla U-Net	92.67	92.15	92.67	91.78
Dense U-Net	91.19	91.57	91.67	91.56
U-Net 3+	93.46	93.67	93.87	93.99

**Table 6:** A comparative of different segmentation models using only IGAN pre-augmentation.

Model	Dice Score	Accuracy	Precision	Recall
U-Net	92.67	92.67	92.65	92.19
Multi residual U-Net	93.67	93.45	93.59	93.51
U-Net++	93.56	93.78	93.16	93.87
Attention U-Net++	94.69	94.69	94.25	94.65
Vanilla U-Net	93.56	93.45	93.25	93.45
Dense U-Net	92.67	92.68	92.48	92.67
U-Net 3+	95.49	95.67	95.59	95.68

## Data Availability

Furnished on request.
